# Inhibition of Cdk5 Ameliorates Skeletal Bone Loss in Glucocorticoid-Treated Mice

**DOI:** 10.3390/biomedicines10020404

**Published:** 2022-02-08

**Authors:** Benjamin Thilo Krüger, Lena Steppe, Sabine Vettorazzi, Melanie Haffner-Luntzer, Sooyeon Lee, Ann-Kristin Dorn, Anita Ignatius, Jan Tuckermann, Mubashir Ahmad

**Affiliations:** 1 Institute of Orthopedic Research and Biomechanics, Ulm University, Helmholtzstrasse 14, 89081 Ulm, Germany; benjamin.krueger@uni-ulm.de (B.T.K.); lena.steppe@uni-ulm.de (L.S.); melanie.haffner-luntzer@uni-ulm.de (M.H.-L.); 2 Institute of Comparative Molecular Endocrinology (CME), Ulm University, Helmholtzstrasse 8/1, 89081 Ulm, Germany; sabine.vettorazzi@uni-ulm.de (S.V.); sooyeon.lee@uni-ulm.de (S.L.); ann.kristin.picke@gmail.com (A.-K.D.)

**Keywords:** osteoblasts, osteoclasts, osteocytes, bone, cyclin-dependent kinase 5, roscovitine, glucocorticoids, glucocorticoid-induced osteoporosis, fracture healing

## Abstract

Glucocorticoids (GCs) are widely used to treat inflammatory diseases. However, their long-term use leads to glucocorticoid-induced osteoporosis, increasing morbidity and mortality. Both anabolic and anti-resorptive drugs are used to counteract GC-induced bone loss, however, they are expensive and/or have major side effects. Therefore, identifying new targets for cost-effective, small-molecule inhibitors is essential. We recently identified cyclin-dependent kinase 5 (Cdk5) as a suppressor of osteoblast differentiation and showed that its inhibition with roscovitine promoted osteoblastogenesis, thus improving the skeletal bone mass and fracture healing. Here, we assessed whether Cdk5 knockdown or inhibition could also reverse the GC-mediated suppression of osteoblast differentiation, bone loss, and fracture healing. We first demonstrated that *Cdk5* silencing abolished the dexamethasone (Dex)-induced downregulation of alkaline phosphatase (Alp) activity, osteoblast-specific marker gene expression (*Runx2*, *Sp7*, *Alpl*, and *Bglap*), and mineralization. Similarly, Cdk5 inhibition rescued Dex-induced suppression of Alp activity. We further demonstrated that Cdk5 inhibition reversed prednisolone (Pred)-induced bone loss in mice, due to reduced osteoclastogenesis rather than improved osteoblastogenesis. Moreover, we revealed that Cdk5 inhibition failed to improve Pred-mediated impaired fracture healing. Taken together, we demonstrated that Cdk5 inhibition with roscovitine ameliorated GC-mediated bone loss but did not reverse GC-induced compromised fracture healing in mice.

## 1. Introduction

Glucocorticoids (GCs) are potent anti-inflammatory agents to treat inflammatory diseases including rheumatoid arthritis, chronic obstructive pulmonary disease, and inflammatory bowel disease [1,2,3,4,5]. However, long-term GC therapy is associated with glucocorticoid-induced osteoporosis (GIO), the most prevalent form of secondary osteoporosis [6,7]. The adverse effects of long-term GC therapy on bone increase the risk for fragility fractures in a dose-dependent manner, which exacerbates the cause of disability and mortality [8,9]. Supraphysiological GC levels reduce bone mass primarily by decreasing osteoblast function and bone formation [10,11,12,13,14,15,16,17]. In addition, high-dose GCs increase the receptor activator of nuclear factor kappa-B ligand (RANKL) and reduce osteoprotegerin (OPG) levels, thus inducing osteoclastogenesis and bone resorption [14,18,19].

In addition to their well-known negative effects on bone mass, GCs also influence the complex process of bone fracture healing that includes the consecutive phases of inflammation, soft and hard callus formation, and remodeling until the restoration of the original bone structure and shape [20]. Recently, endogenous GC signaling was shown to be essential for effective bone regeneration [21,22,23]. However, exogenous treatment with supraphysiological GC doses significantly reduces bone formation after fracture and the quality of the newly formed bone [24,25,26,27,28].

Current treatment regimens for GIO include anti-resorptive and osteoanabolic drugs, including oral and intravenous bisphosphonates (BPs), denosumab, human parathyroid hormone 1–34 (hPTH 1–34), and supplementation of calcium and vitamin D [29,30,31,32,33,34,35,36,37]. Indeed, these treatments have proven to ameliorate bone loss in GIO and reduce the burden of GC-mediated fractures [38,39,40]. Similarly, BP treatment reversed GC-mediated, compromised fracture healing in a pre-clinical study in rats [41]. Contrarily, osteoanabolic therapy with hPTH 1–34 failed to improve fracture healing in GC-treated mice [42]. Although some anti-osteoporotic drugs show a promising outcome in both GC-mediated GIO and impaired fracture healing, they either are expensive or have major side effects [43,44,45,46,47]. Therefore, the identification of targets for cost-effective, small-molecule inhibitors with minimal side effects is of utmost importance.

Cyclin-dependent kinase 5 (Cdk5), a member of the proline-directed serine/threonine cyclin-dependent kinase family, largely controls a number of neuronal functions and is known to be a major player in the pathogenesis of neurodegenerative diseases [48,49,50,51,52]. Recently, we identified Cdk5 as a strong suppressor of osteoblast differentiation and showed that its inhibition with the small-molecule inhibitor, roscovitine, increased bone mass and improved fracture healing in skeletally healthy mice [53]. On the basis of these observations, we here investigated whether Cdk5 inhibition with roscovitine could also reverse GC-induced bone loss and compromised fracture healing.

## 2. Materials and Methods

### 2.1. Isolation of Primary Murine Calvarial Osteoblasts

Primary calvarial osteoblast isolation was performed using neonatal mouse calvaria of 2–5-day-old pups as previously described [54,55]. Briefly, the calvariae were isolated in 1 mL digestion solution (0.2% *w*/*v* each of collagenase A and dispase II (Roche, Basel, Switzerland)) and incubated at 37 °C for 10 min at 700 rpm on a shaker. The digestion was performed five times, and all but the first supernatant were collected in 15 mL falcons containing 500 µL fetal bovine serum (GE Healthcare, Chicago, IL, USA). The collected supernatant was centrifuged (252× *g*; 5 min; room temperature (RT)), resuspended in 3 mL complete α-minimum essential medium (ThermoFisher Scientific, Waltham, MA, USA) and placed in a six-well plate. Following overnight incubation (37 °C; 5% CO_2_), the medium was replaced with a fresh medium. The experiments were performed at an 80% confluency as previously described [53,54].

### 2.2. Small Interfering RNA (siRNA) Transfection

SMARTpool non-targeting siRNA control (si*NT*) and *Cdk5*-specific siRNA (si*Cdk5*) were purchased from Horizon Discovery (Waterbeach, UK). The transfection was performed using a final concentration of 20 nM siRNA with 0.125% Lipofectamine RNAiMAX transfection reagent (Life Technologies, ThermoFisher Scientific, Waltham, MA, USA), as previously described [54]. The siRNA sequences used are listed in Table 1.

### 2.3. Murine Primary Calvarial Osteoblast Differentiation

For experiments with primary murine calvarial osteoblasts, the cells were seeded at a confluency of 12,000 cells/cm^2^. After 48 h, the cells were differentiated by adding an osteogenic induction medium (100 µg/mL (+)-sodium L-ascorbate and 5 mM β-glycerophosphate (Sigma-Aldrich, St. Louis, MO, USA)). The osteogenic induction medium was refreshed every third day.

Treatment with roscovitine (0.16 µM) (Selleckchem, Houston, TX, USA) was performed in an osteogenic induction medium, as previously described [53]. An ethanol vehicle was used at a concentration of 0.01% as a control. Treatment with roscovitine was performed every third day until the termination of the experiment.

### 2.4. PrestoBlue Cell Viability Assay

Cell viability was tested using the PrestoBlue cell viability reagent (Thermo Fisher Scientific, Waltham, MA, USA) according to the manufacturer’s instructions. Briefly, 3.2 mL of the medium were removed from 60 mm dishes, followed by the addition of 200 µL cell viability reagent. In parallel, in a 96-well plate, the medium and the cell viability reagent were mixed in a volume ratio of 9:1 to obtain a final volume of 100 µL that served as a blank. After incubating the plates (37 °C; 5% CO_2_) for 30 min, 100 µL aliquots were measured against the blank at a 570 nm absorbance using a Dynex Opsys MR microplate reader (Aspect Scientific, Cheshire, UK).

### 2.5. Alkaline Phosphatase (Alp) and Alizarin Red S (ARS) Staining

For quantitative Alp, primary murine calvarial osteoblasts were reversely transfected and seeded in a 384-well plate. The cells were differentiated by adding an osteogenic induction medium, fixed and stained with ELF 97 (Thermo Fisher Scientific, Waltham, MA, USA) for Alp, and DRAQ5 (BioStatus Ltd., Loughborough, UK) for nuclei, and were analyzed as previously described [54]. For Cdk5 inhibition in vitro, primary murine calvarial osteoblasts were treated with either a vehicle or roscovitine (0.16 µM) for six days as previously described [53].

For qualitative and quantitative ARS (Sigma-Aldrich, St. Louis, MO, USA) staining, the primary calvarial osteoblasts were fixed with 4% paraformaldehyde (PFA) (10 min; RT) and incubated with 1% ARS (1 h; RT). The excessive ARS was removed by washing with 1× phosphate-buffered saline (PBS), and stereomicroscopic images were obtained using a Leica microscope (Leica Camera AG, Wetzlar, Germany). For quantitative evaluation, the ARS stain was extracted by the acetic acid method and neutralization with ammonium hydroxide as previously described [56]. The colorimetric measurement of the extracted solution was performed using a Dynex Opsys MR microplate reader (Dynex Technologies GmbH, Denkendorf, Germany) at an absorption of 405 nm. Finally, the measurements were normalized to the cell viability.

### 2.6. RNA Isolation, cDNA Synthesis, and Real-Time Polymerase Chain Reaction (RT-PCR)

RNA isolation was performed using a RNeasy kit (Qiagen, Hilden, Germany) according to the manufacturer’s instructions. Following the isolation procedure, 1 µg RNA was used for reverse transcription using a RevertAid H Minus reverse transcriptase kit (Fermentas, Waltham, MA, USA) or a high-capacity cDNA kit (Thermo Fisher Scientific, Waltham, MA, USA). RT-PCR was performed using a ViiA 7 PCR system (Applied Biosystems, Waltham, MA, USA), and relative mRNA concentrations were normalized to β-actin (*Actb*) using the ΔΔCt method. The mouse primer sequences used in this study are listed in Table 2.

### 2.7. Protein Isolation, Quantification, and Western Blotting

The whole cell protein was isolated using a radioimmunoprecipitation assay buffer and quantified using a Pierce BCA protein assay kit (ThermoFisher Scientific, Waltham, MA, USA). We used 30 µg protein from each sample and subjected them to western blotting as previously described [54]. We used antibodies against Cdk5 (Cell Signaling Technology, Danvers, MA, USA) and α-tubulin (Sigma-Aldrich, St. Louis, MO, USA). The band intensities of western blots were quantified using Fiji ImageJ [57].

### 2.8. Animals

All mouse experiments were in compliance with the international regulations for the care and use of laboratory animals with the approval of the local ethical committee (No.1245/1402 Regierungspräsidium Tübingen, Germany). Eleven- and 13-week-old wild-type female and male BALB/cAnNCrl mice were separately maintained in single house units under controlled standard conditions (Makrolon type II long; 530 cm^2^), with a 12 h light and dark circadian rhythm with water and food (Ssniff, Soest, Germany) ad libitum at 23 °C and a humidity of 55% ± 10%) in a pathogen-free animal facility at Ulm University. To reduce the number of mice, we followed the replace, reduce, refine (3Rs) principle for the ethical use of animals. Therefore, we here used the control group (sham/vehicle) derived from our previous study [53], as these experiments were run in parallel.

### 2.9. GIO Model

Eleven-week-old wild-type female BALB/cAnNCrl mice (Charles River Laboratories, Wilmington, MA, USA) received a subcutaneous slow-release sham- or prednisolone (Pred)-pellet (12 mg/kg/day) (Innovative Research of America, Sarasota, FL, USA) at the neck, as previously described [58]. The mice were injected intraperitoneally (i.p.) with either a vehicle (5% dimethyl sulfoxide (DMSO), 10% kolliphor EL (Sigma-Aldrich, Taufkirchen, Germany), 85% 1× PBS), or roscovitine (150 mg/kg) (LC Laboratories, Woburn, MA, USA), three times a week for two weeks, as previously described [53]. The surgery was performed under general anesthesia (2 volume percent (vol%) isoflurane (Baxter, Unterschleißheim, Germany)). After two weeks, the mice were euthanized by an overdose of isoflurane, and the femora were collected for further analyses.

### 2.10. Fracture Healing Model

To study fracture healing, we used a standardized osteotomy model as previously described [59]. The osteotomy was performed at the right femur diaphysis of 12-week-old wild-type male BALB/cAnNCrl mice (Charles River Laboratories, Wilmington, MA, USA). All surgeries were performed under general anesthesia (2 vol% isoflurane). The mice first received clindamycin (45 mg/kg) (MIP Pharma Holding GmbH, Blieskastel, Germany) as an anti-infective treatment just prior to the surgery, and tramadol-hydrochloride in the drinking water (25 mg/L) (Grünenthal, Aachen, Germany) as pain medication one day prior until day three post-surgery, as previously described [59]. During the procedure, the right femur was exposed and stabilized using a semi-rigid external fixator with an axial stiffness of 3 N/mm and four mini-Schanz screws (RISystem, Davos, Switzerland). A midshaft osteotomy was performed using a gigli wire saw (0.4 mm in diameter). Additionally, the mice received a subcutaneous slow-release sham- or prednisolone-pellet (12 mg/kg/day) [10,58] and were further injected i.p. with either a vehicle (5% DMSO, 10% kolliphor EL, and 85% 1× PBS) or roscovitine (150 mg/kg) (Selleckchem, Houston, TX, USA), every second day for 14 or 23 days. After the respective time-points, the mice were euthanized by an overdose of isoflurane, and osteotomized femora were collected for further analyses.

### 2.11. Biomechanical Testing of the Fractured Femurs

At day 23 post-surgery, fractured femurs were subjected to biomechanical testing using a non-destructive three-point bending test in a universal material testing machine, Zwick Z10 (Zwick Roell, Ulm, Germany), to assess the functional healing outcome, as previously described [59]. Briefly, after the fixation of the proximal end of the femur into a hinge joint of the testing setup, an increasing load up to a maximum of 2 N was applied to the middle of the callus (2 mm/min). Flexural rigidity was calculated using the slope (*k*) of the load–displacement curve in the linear region [59].

### 2.12. Microcomputed Tomography (µCT) Analysis

Intact femurs were analyzed using a high-resolution µCT Skyscan 1176 scanner (Bruker Corporation, Billerica, MA, USA). Images at a 9 µm voxel resolution were acquired using a 50 kV X-ray voltage, a 200 µA current, and a 0.5 mm aluminum filter with a 1° rotation step. Following reconstruction using NRecon and DataViewer (Bruker Corporation, Billerica, MA, USA), the trabecular and cortical bone analysis was performed at the 0.215 and 1.935 mm proximal of the growth plate using 1.29- and 0.43-mm regions of interest, respectively. The structural analysis was performed using the CTAn software (Bruker Corporation, Billerica, MA, USA). Three-dimensional images were created using CTVox (Bruker Corporation, Billerica, MA, USA).

In the fractured femora, the region of interest was set as the periosteal callus between both inner pinholes. The bone volume fraction (BV/TV) was measured under a global threshold of 642 mg hydroxyapatite/cm^3^ as previously described [60]. All the measurements were performed in accordance with the guidelines of the American Society for Bone and Mineral Research (ASBMR) [61].

### 2.13. Histomorphometry

For static bone histomorphometry, femurs were isolated, fixed in 4% PFA for three days and decalcified with 15% ethylenediamine tetraacetic acid for 10 days followed by paraffin embedding, as previously described [53,62]. Femur sections of seven micrometers were cut and stained for tartrate-resistant acid phosphatase (TRAP) as previously described [63]. Osteoclasts were counted as multinucleated TRAP-positive cells, whereas osteoblasts were counted as cubic-shaped cells with visible cytoplasm. The following cellular parameters were measured: osteoclast surface per bone surface (Oc.S/BS), osteoclast number per bone perimeter (Oc.N/B.Pm), osteoblast surface per bone surface (Ob.S/BS), and osteoblast number per bone perimeter (Ob.N/B.Pm), using Osteomeasure software (Osteometrics, Decatur, IL, USA) according to the ASBMR guidelines [64,65].

Fractured femora were stained with Safranin-O/Fast Green to analyze the callus, bone, cartilage, and soft tissue areas, using Leica LASX image analysis software (Leica, Heerbrugg, Switzerland).

For dynamic bone histomorphometry, the mice received i.p. calcein injections nine and two days prior to sacrifice as previously described [63,64,66]. Femurs were fixed in 4% PFA and embedded in methyl methacrylate as previously described [64,66]. Femur sections of seven micrometers were cut to determine the bone formation rate (BFR) and the mineral apposition rate (MAR) using the Osteomeasure software (Osteometrics, Decatur, IL, USA).

### 2.14. N-Terminal Propeptide of Type I Procollagen (PINP) and C-Terminal Telopeptides of Type I Collagen (CTX-I) ELISAs

The blood of the mice was collected in heparin-coated tubes and centrifuged at 2000× *g* for 10 min at RT to collect the plasma. ELISAs for PINP and CTX-I (Immunodiagnostic Systems, East Boldon, UK) were performed according to the manufacturer’s instructions.

### 2.15. Statistical Analysis

Data are represented as box and whisker plots with the minimum to the maximum as well as superimposing all of the data points. Statistical differences between the groups were determined by ordinary one-way ANOVA using Tukey’s multiple comparison test. A *p*-value less than 0.05 was considered to be statistically significantly different (* *p* < 0.05, ** *p* < 0.01, *** *p* < 0.001).

## 3. Results

### 3.1. Cdk5 Deletion or Inhibition Antagonizes Suppressive Effects of GCs on Osteoblast Differentiation and Mineralization

**Figure 1:**
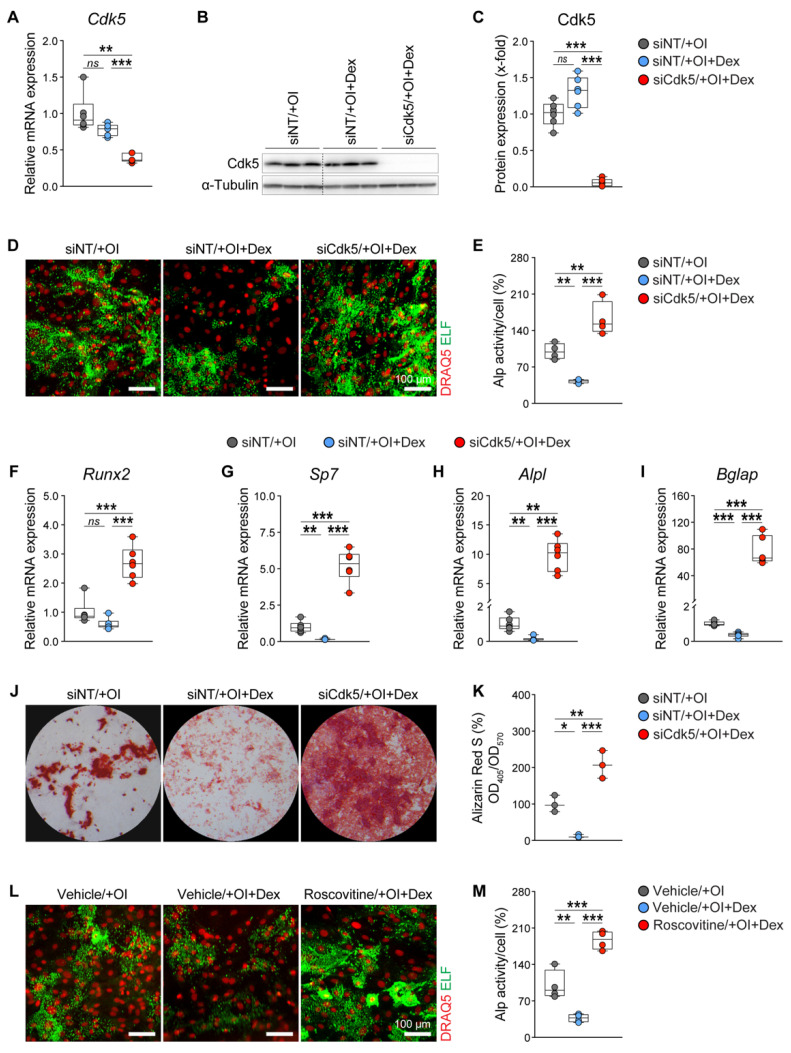
Glucocorticoid (GC)-mediated suppression of osteoblast differentiation and mineralization is reversed by Cdk5 deletion or inhibition. Primary murine calvarial osteoblasts were transfected either with non-targeting siRNA (si*NT*) or *Cdk5*-specific siRNA (si*Cdk5*) for eight days in the presence or absence of 1 µM dexamethasone (Dex): (**A**) *Cdk5* mRNA expression (n = 6); (**B**,**C**) Cdk5 protein levels and its quantification (the siCdk5/+OI group was cropped out as shown with the dotted line. For the original, see Figure S1) (n = 6); (**D**,**E**) representative microscopic images of nuclear (DRAQ5 in red) and Alp (ELF 97 in green) staining upon different treatments and their quantification (n = 4); (**F**–**I**) mRNA expression of osteoblast-specific marker genes *Runx2*, *Sp7*, *Alpl*, and *Bglap* (n = 6); (**J**,**K**) qualitative and quantitative Alizarin Red S staining in primary murine calvarial osteoblasts after 20 days of transfection with si*NT* or si*Cdk5*, in the presence or absence of 1 µM Dex (n = 3); (**L**,**M**) representative microscopic images of nuclear (red) and Alp (green) staining upon different treatments and their quantification in primary murine calvarial osteoblasts after six days of treatment with a vehicle (EtOH) or roscovitine (0.16 µM), in the presence or absence of 1 µM Dex (n = 4). Data are represented as box and whisker plots with the minimum to the maximum as well as superimposing all of the data points. Statistical differences between two groups were determined by one-way ANOVA and Tukey’s test. * *p* < 0.05; ** *p* < 0.01; *** *p* < 0.001, ns: no significance.

Dexamethasone (Dex), a widely used synthetic GC, was shown to suppress osteogenic differentiation and mineralization [10,67,68]. To investigate whether *Cdk5* depletion can affect Dex-mediated osteoblast suppression, we transfected primary murine calvarial osteoblasts with non-targeting siRNA (si*NT*) or *Cdk5*-specific siRNA (si*Cdk5*) and induced them into the osteogenic lineage in the presence or absence of 1 µM Dex. First, we found that *Cdk5* mRNA and protein expression was not regulated by Dex treatment and that it was significantly reduced upon siRNA knockdown (Figure 1A–C). We further showed that the Dex treatment significantly reduced cellular Alp activity, while the co-treatment with si*Cdk5* abrogated the GC-induced suppression of Alp activity (Figure 1D,E). Furthermore, Dex treatment reduced the expression of osteoblast-specific transcription factors and marker genes such as *Runx2*, *Sp7*, *Alpl*, and *Bglap*, whereas the co-treatment with si*Cdk5* rescued their expression (Figure 1F–I). Moreover, as demonstrated by Alizarin red staining, in vitro matrix mineralization was significantly reduced by Dex treatment, whereas this was ameliorated by co-treatment with si*Cdk5* (Figure 1J,K).

Recently, we reported that Cdk5 inhibition with roscovitine enhances osteoblast differentiation and bone formation [53]. Therefore, to determine whether Cdk5 inhibition with roscovitine can also affect Dex-induced osteoblast suppression, we treated primary murine calvarial osteoblasts with either a vehicle or roscovitine (0.16 µM) in the presence or absence of 1 µM Dex. Consistent with the siRNA data (Figure 1A–K), we showed that the roscovitine treatment reversed the GC-mediated suppression of osteoblast function (Figure 1L,M). Taken together, these results confirmed that Cdk5 deletion or inhibition counteracted the Dex-mediated suppression of osteoblast differentiation and mineralization in vitro.

### 3.2. Cdk5 Inhibition Antagonizes GC-Mediated Bone Loss by Reducing Osteoclastogenesis

**Figure 2:**
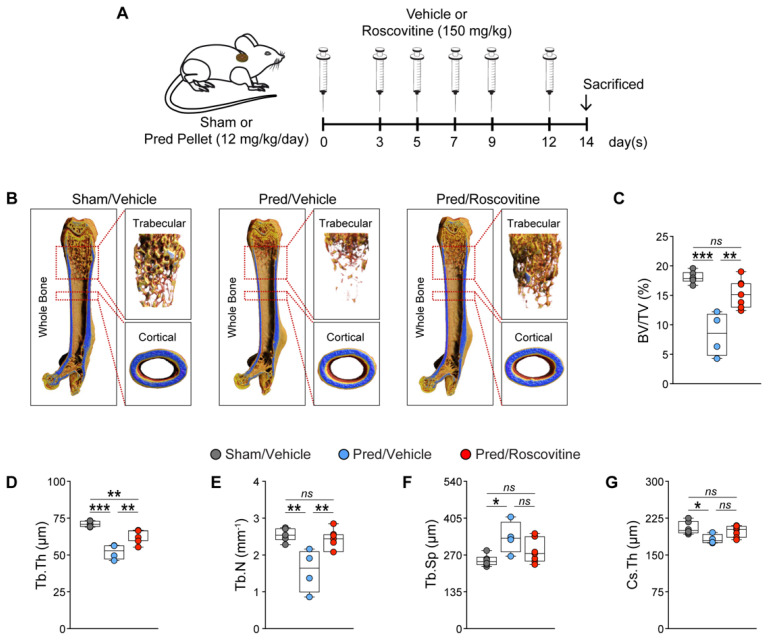
Inhibition of Cdk5 with roscovitine ameliorates GC-mediated loss of bone mass in mice. (**A**) Experimental setup for the implantation of a sham or prednisolone (Pred)-pellet, followed by treatment with a vehicle or roscovitine (intraperitoneally (i.p.); 150 mg/kg; three times per week) for 14 days. (**B**) Representative micro-computed tomography (µCT) images of whole, trabecular and cortical bone upon different treatments. The calculated femoral trabecular and cortical parameters include the following: (**C**) bone volume fraction (BV/TV; %); (**D**) trabecular thickness (Tb.Th; µm); (**E**) trabecular number (Tb.N; mm^−1^); (**F**) trabecular separation (Tb.Sp; µm); and (**G**) cross-sectional thickness (Cs.Th; µm) (n = 4–6). Data are represented as box and whisker plots with the minimum to the maximum as well as superimposing all of the data points. Statistical differences between two groups were determined by one-way ANOVA and Tukey’s test. * *p* < 0.05; ** *p* < 0.01; *** *p* < 0.001, ns: no significance.

To investigate whether roscovitine treatment affects GC-mediated bone loss in vivo, we implanted a sham or Pred pellet in wild-type mice and treated them with either a vehicle or roscovitine for 14 days (Figure 2A). The µCT analysis revealed a significant loss of bone mass in the distal femurs of Pred-treated mice. This was due to decreases in trabecular thickness and number, but increased trabecular separation (Figure 2B–F). Importantly, the roscovitine treatment abrogated these Pred-mediated deleterious effects on bone mass (Figure 2B–F). In addition, the crossectional thickness was significantly reduced in the Pred-treated mice, which was reversed by roscovitine co-treatment (Figure 2G).

**Figure 3:**
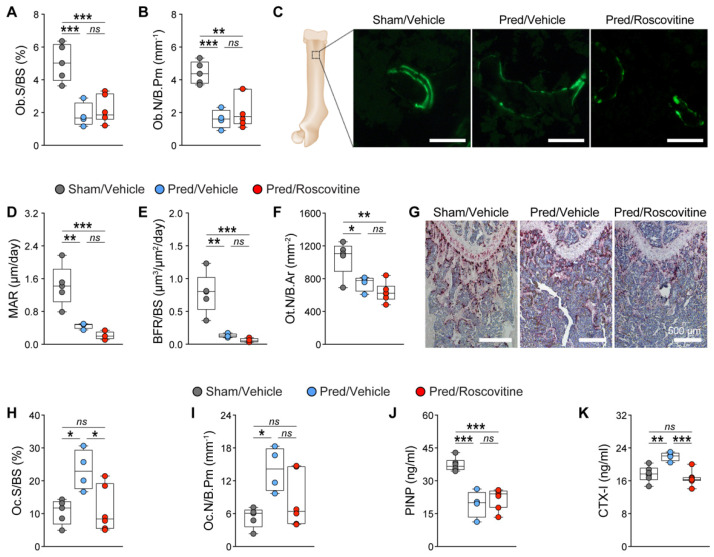
Inhibition of Cdk5 with roscovitine alleviates GC-mediated bone loss by reducing osteoclastogenesis. Static and dynamic bone histomorphometry were performed from different treatments, and the following parameters were calculated from trabecular bone: (**A**) osteoblast surface per bone surface (Ob.S/BS; %); (**B**) osteoblast number per bone parameter (Ob.N/B.Pm; mm^−1^); (**C**) representative images of dual calcein labeling (green) (scale: 100 µm); **(D**) mineral apposition rate (MAR; µm/day); (**E**) bone formation rate (BFR/BS; µm^3^/µm^2^/day); (**F**) osteocyte number per bone area (Ot.N/B.Ar; mm^−2^); (**G**) representative images of tartrate-resistant acid phosphatase (TRAP) staining for osteoclasts (purple) (scale: 500 µm); (**H**) osteoclast surface per bone surface (Oc.S/BS; %); and (**I**) osteoclast number per bone parameter (Oc.N/B.Pm; mm^−1^) (n = 4–6). Analysis of bone formation and resorption markers: (**J**) N-terminal propeptide of type I procollagen (PINP; ng/mL); and (**K**) C-terminal telopeptides of type I collagen (CTX-I; ng/mL) (n = 4–6). Data are represented as box and whisker plots with the minimum to the maximum as well as superimposing all of the data points. Statistical differences between two groups were determined by one-way ANOVA and Tukey’s test. * *p* < 0.05; ** *p* < 0.01; *** *p* < 0.001, ns: no significance.

To determine the effect of the roscovitine treatment on bone cells in vivo, we performed static and dynamic bone histomorphometry. We observed significant decreases in the osteoblast surface and number in both trabecular and cortical bone in Pred-treated mice, which was not reversed by the roscovitine co-treatment (Figure 3A,B; Figure S2A,B). The dynamic bone histomorphometry confirmed these findings: MAR and BFR were reduced in Pred-treated mice, and this was not reversed by co-treatment with roscovitine (Figure 3C–E; Figure S2C–E). In addition, the decreased osteocyte number induced by the Pred treatment was not rescued by synergistic treatment with roscovitine (Figure 3F; Figure S2F). By contrast, the osteoclast surface and number, which were significantly increased after the Pred treatment in both trabecular and cortical bone, were reduced to control levels by the roscovitine co-treatment (Figure 3G–I; Figure S2G,H). These results were further confirmed by reduced plasma PINP levels and increased CTX-I levels in the Pred-treated mice, indicating reduced bone formation and increased bone resorption, respectively (Figure 3J,K). However, only the CTX-I levels returned to control levels in mice that received both Pred and roscovitine treatments (Figure 3J,K).

These results suggested that the rescue of GC-mediated bone loss in the combinatorial treatment group was due to a reduced osteoclastogenesis rather than an improved osteoblastogenesis.

Because osteoclastogenesis is indirectly regulated by osteoblasts via the expression of *Rankl* and *Opg* [69,70], we also investigated whether the *Rankl*/*Opg* axis is modulated in primary murine calvarial osteoblasts by Dex and si*Cdk5*. Indeed, we observed a significant increase in the *Rankl*/*Opg* expression ratio upon Dex treatment, which was reduced by the co-treatment with si*Cdk5* (Figure S3), suggesting a possible mechanism of the reduction in GC-induced osteoclastogenesis upon Cdk5 inhibition in vivo.

In conclusion, we demonstrated that GC-mediated bone loss is ameliorated by Cdk5 inhibition with roscovitine through a reduction in osteoclastogenesis.

### 3.3. Cdk5 Inhibition Does Not Reverse GC-Mediated Impaired Fracture Healing

**Figure 4:**
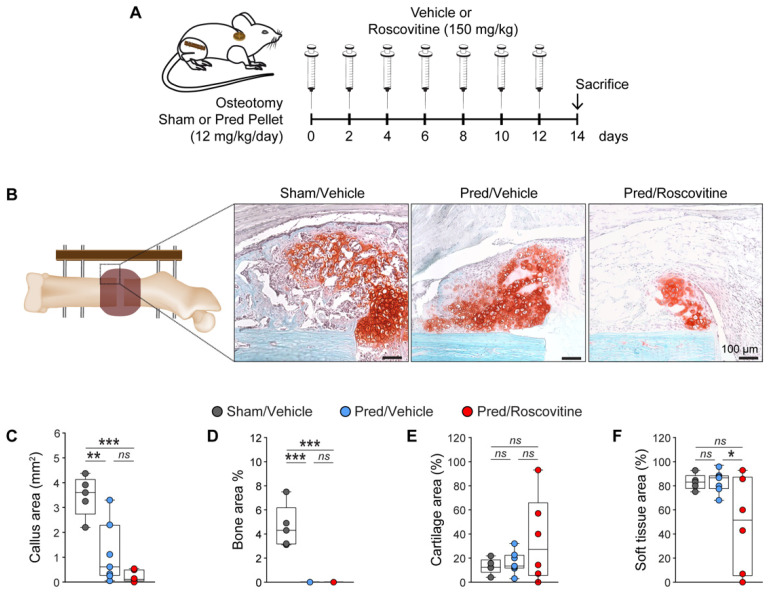
GC-mediated delayed fracture healing is not reversed by Cdk5 inhibition with roscovitine during soft callus formation. (**A**) Experimental setup for the osteotomy and implantation of a sham or Pred pellet, followed by treatment with a vehicle or roscovitine (i.p.; 150 mg/kg; every second day) for 14 days, to evaluate soft callus formation. (**B**) Representative images of Safranin O/Fast Green staining of the fracture callus after different treatments (scale: 100 µm). The following parameters were calculated: (**C**) callus area (mm^2^); (**D**) bone area (%); (**E**) cartilage area (%); and (**F**) soft tissue area (%) (n = 5–7). Data are represented as box and whisker plots with the minimum to maximum as well as superimposing all of the data points. Statistical differences between two groups were determined by one-way ANOVA and Tukey’s test. * *p* < 0.05; ** *p* < 0.01; *** *p* < 0.001, ns: no significance.

To investigate GC-mediated impaired fracture healing under roscovitine treatment, we performed an open femur osteotomy, implanted a sham or Pred pellet and treated the mice with either a vehicle or roscovitine for 14 days (Figure 4A). Our results showed that after 14 days, a time point of extensive endochondral bone formation in murine fracture healing, the Pred treatment decreased the callus size and the bone area in the fracture callus, which were not reversed by the combinatorial treatment with roscovitine (Figure 4B–D). In addition, we did not observe any changes in cartilage or soft tissue areas upon Pred treatment alone or in combination with roscovitine (Figure 4B,E,F).

**Figure 5:**
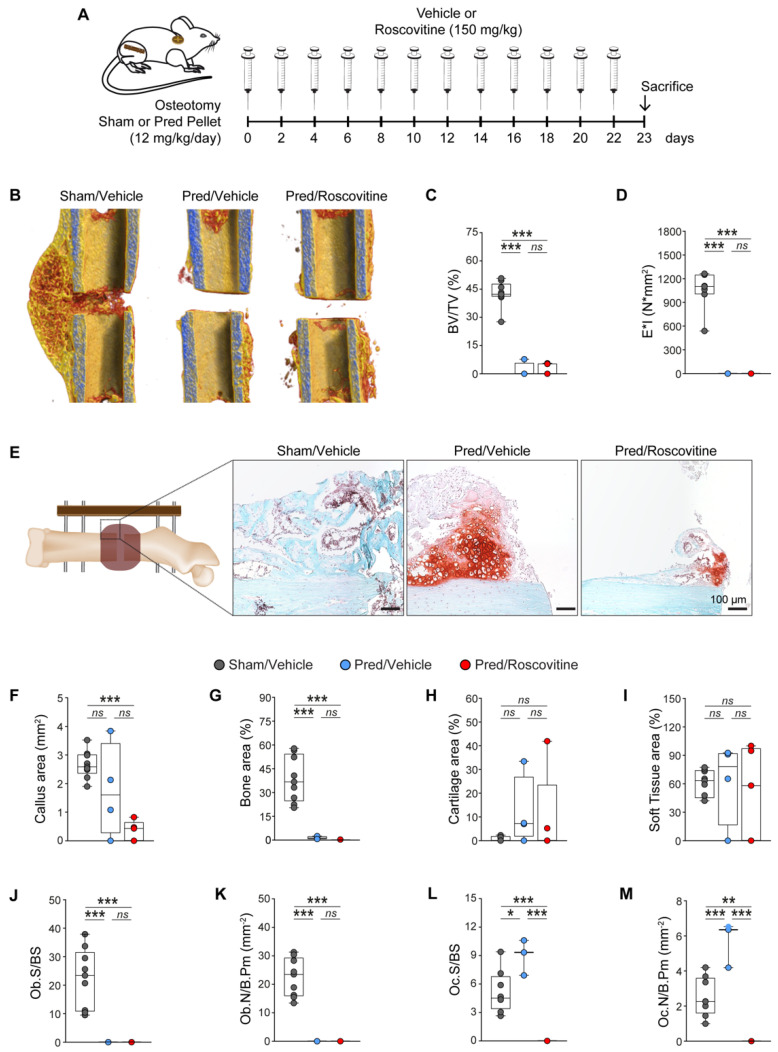
GC treatment delays fracture healing through decreased osteoblastogenesis, which could not be rescued by roscovitine application. (**A**) Experimental setup for the osteotomy and implantation of a sham or Pred pellet, followed by treatment with a vehicle or roscovitine (i.p.; 150 mg/kg; every second day) for 23 days, to evaluate hard callus formation. (**B**) Representative µCT images of fractured femurs from different treatments. The calculated parameters include the following: (**C**) bone volume fraction (BV/TV; %) (n = 4–9); and (**D**) bending stiffness (E*I; N/mm^2^) (n = 4–7). (**E**) Representative images of the Safranin O/Fast Green staining of fracture callus after different treatments (scale: 100 µm). The following parameters were calculated: (**F**) callus area (mm^2^); (**G**) bone area (%); (**H**) cartilage area (%); (**I**) soft tissue area (%); (**J**) osteoblast surface per bone surface (Ob.S/BS; %); (**K**) osteoblast number per bone parameter (Ob.N/B.Pm; mm^−1^) (n = 4–9); (**L**) osteoclast surface per bone surface (Oc.S/BS; %); and (**M**), osteoclast number per bone parameter (Oc.N/B.Pm; mm^−1^) (n = 4–8). Data are represented as box and whisker plots with the minimum to the maximum as well as superimposing all of the data points. Statistical differences between two groups were determined by one-way ANOVA and Tukey’s test. * *p* < 0.05; ** *p* < 0.01; *** *p* < 0.001, ns: no significance.

At a later healing stage of 23 days, the Pred treatment (Figure 5A) resulted in significantly impaired hard callus formation, as shown by a greatly reduced BV/TV and bending stiffness, which was not reversed by the combinative treatment with roscovitine (Figure 5B–D). These findings were further confirmed by the histological evaluation. While the callus area was not significantly affected, the bone area was significantly reduced in the Pred-treated mice compared to the control group. The co-treatment with Pred and roscovitine significantly reduced both the callus size and the bone content (Figure 5E–G), while the cartilage and soft tissue areas remained unaffected by the treatments (Figure 5E,H,I). The Pred treatment significantly decreased the osteoblast number and surface, whereas the osteoclast number and surface were significantly increased in the newly formed bone of the fracture callus (Figure 5J–M). In the combinatorial treatment, the bone formation in the callus was abrogated, and no osteoblasts and osteoclasts were present (Figure 5G,J–M).

In summary, we conclude that Cdk5 inhibition with roscovitine was unable to ameliorate the deleterious effects of GCs on bone fracture healing.

## 4. Discussion

Common side effects of GC treatment in the context of bone biology are GIO and impaired fracture healing [1,13,27,28,71]. Recently, we identified Cdk5 as a promising target to increase osteoblast differentiation and bone mass and improve fracture healing in skeletally healthy mice [53]. Here, we assessed whether the pharmacological inhibition of Cdk5 with roscovitine has the potential to ameliorate the adverse effects of GCs on bone and in impairing fracture healing. Indeed, Cdk5 inhibition rescued GC-induced skeletal bone loss through reduced osteoclastogenesis, however; it did not reverse GC-mediated compromised fracture healing.

Cdk5 is known to be involved in neuronal differentiation [72], and its aberrant activity contributes to the pathogenesis of neurodegenerative disorders, including amyotrophic lateral sclerosis and Huntington’s, Alzheimer’s, and Parkinson’s diseases [50,51,52]. Additionally, there is growing evidence that Cdk5 plays a role in T cell activation and cancer biology [73,74,75]. Recently, our group reported for the first time a crucial role of Cdk5 in bone biology [53]. Cdk5 strongly suppresses osteoblast differentiation through the mitogen-activated protein kinase pathway, and Cdk5 inhibition with the small-molecule inhibitor, roscovitine, induces osteoanabolic effects on bone mass and formation during fracture healing in skeletally healthy mice [53]. On the basis of these findings, we here determined whether Cdk5 inhibition has the potential to counteract GC-induced bone loss and impaired fracture healing.

It is well established that high-dose GC treatment inhibits osteoblast differentiation and mineralization in vitro and reduces trabecular and cortical bone mass in vivo [1,3,10,16,17,19,67,68,76,77,78,79]. This GC-mediated bone loss phenotype is mainly attributed to decreased osteoblastogenesis and osteocyte number that subsequently reduce bone formation, but also to increased osteoclastogenesis, which consequently enhances bone resorption [10,19,76,77,78,80]. In the present study, we confirmed the negative effects of high-dose GCs both in vitro and in vivo.

Our in vitro results showed that Cdk5 deletion or inhibition with roscovitine not only completely abolished the GC-mediated detrimental effects on osteoblast differentiation, but even increased osteoblast activity compared to untreated cells. This effect can be explained by a possible crosstalk between GC- and Cdk5-regulated pathways. For example, it is well-known that the extracellular signal-regulated kinase (Erk1/2) pathway is crucial for osteoblastogenesis [81], and exogenous GCs have been shown to inhibit this pathway [82]. Moreover, we recently reported that Cdk5 depletion in osteoblasts activates the Erk1/2 pathway [53], suggesting one of the possible mechanisms through which Cdk5 depletion counteracts the GC-mediated inhibition of the Erk1/2 pathway and consequently the osteoblast differentiation.

Importantly, our in vivo results demonstrated that Cdk5 inhibition with roscovitine ameliorated GC-mediated bone loss in mice. Of note, we here observed that Cdk5 inhibition prevented GC-mediated bone loss through a reduction of osteoclastogenesis rather than by promoting osteoblastogenesis and new bone formation. This is in contrast to our previous study with skeletally healthy mice, where we observed a significant osteoanabolic effect of Cdk5 inhibition caused by the induction of osteoblastogenesis [53]. Although this was unexpected, similar findings have been reported with other osteoblast-stimulating drugs under GC therapy. For example, the osteoanabolic effect of intermittent PTH and abaloparatide treatment was blunted by high-dose GCs [77,83]. Obviously, even if our in vitro results implicate a rescue through osteoblastogenesis, osteoblast function cannot completely be reversed by Cdk5 inhibition in the presence of GCs in vivo.

The reduction of osteoclastogenesis observed in GC-treated mice by Cdk5 inhibition could be explained by the modulation of the ratio of *Rankl*/*Opg* expression. Generally, osteoblasts proportionately express *Rankl*, which regulates the differentiation of precursor cells into multinucleated osteoclasts, and *Opg*, a decoy receptor for *Rankl* that protects the skeleton from excessive bone resorption [84,85,86,87]. However, supraphysiological GC doses are known to modulate the *Rankl*/*Opg* axis by upregulating *Rankl* expression levels and downregulating *Opg* expression levels, which consequently promotes osteoclastogenesis and eventually bone resorption [3,19,78]. To this end, we here observed a similar effect on the *Rankl*/*Opg* axis after treating primary murine calvarial osteoblasts with Dex. Interestingly, *Cdk5* deletion reversed this GC-mediated effect on the *Rankl*/*Opg* ratio, suggesting a possible crosstalk between osteoblasts and osteoclasts, which eventually reduces GC-mediated increased osteoclastogenesis in vivo.

Bone fracture healing is a multifactorial process, which involves overlapping phases of inflammation, soft- and hard-callus formation, during which bone is generated by intramembranous and endochondral ossification, and the remodeling of the initially formed woven bone until the original bone structure is restored [88,89,90]. Disruption at any stage of this highly complex healing cascade can delay or even prevent the healing success [20,88]. GCs have a strong effect on many cell types participating in fracture healing, including immune and mesenchymal cells [21,22], and long-term administration is proposed to induce detrimental effects on all stages of fracture repair [27,28]. To determine the role of Cdk5 in GC-mediated impaired fracture healing, we used the same femur fracture model as in our recent study, where we observed osteoanabolic effects of Cdk5 inhibition and improved fracture healing in healthy mice [53]. As expected from the literature, the healing process was considerably impaired upon GC treatment, both at the early and late phases, as indicated by the significantly reduced bone fraction and osteoblast number and activity in the fracture callus [27,28]. Cdk5 inhibition was not able to reverse these negative effects. This supports our observation that in the GIO model, Cdk5 inhibition rescued increased osteoclastogenesis rather than improved osteoblastogenesis. However, there could be additional reasons for the failure of roscovitine to improve GC-induced impaired bone healing. For example, because Cdk5 is known to regulate inflammation [91,92,93,94], its inhibition could possibly exacerbate GC effects on the immune response upon fracture, which is essential for downstream regenerative processes [22,88,89,95]. Another possible reason could be that Cdk5 inhibition or high-dose GCs adversely affects angiogenesis [16,96,97,98,99,100,101], a process that is essential for uneventful fracture healing [89,95].

## 5. Conclusions

In conclusion, our results demonstrated that, even if the pharmacological inhibition of Cdk5 with roscovitine did not reverse GC-induced compromised fracture healing in mice, it ameliorated GC-mediated bone loss in the skeleton. Together with our previous data [53], this indicates that Cdk5 could be a potential therapeutic target to treat GIO. However, further studies are necessary to elucidate the role of Cdk5 in bone, particularly in inflammatory bone disorders.

## Figures and Tables

**Table 1:** Mouse siRNA sequences used in this study.

Gene Symbol	Gene Name	Gene ID	Reverse Primer (5′–3′)
*Non-targeting*	-	-	*UAAGGCUAUGAAGAGAUAC*
			*AUGUAUUGGCCUGUAUUAG*
			*AUGAACGUGAAUUGCUCAA* *UGGUUUACAUGUCGACUAA*
*Cdk5*	*Cyclin-dependent kinase 5*	*12568*	*GGAGAUCUGUCUACUCAAA*
			*UAUAAGCCCUACCCAAUGU* *GCAACGUGCUACAUAGGGA* *CAACAUCCUUGGUGAACGU*

**Table 2:** Oligonucleotide primer sequences from mice used in real-time polymerase chain reaction (RT-PCR).

Gene Symbol	Gene ID	Forward Primer (5′–3′)	Reverse Primer (5′–3′)
*Cdk5*	*12568*	*TGGACCCTGAGATTGTGAAGT*	*GACAGAATCCCAGGCCTTTC*
*Runx2*	*12393*	*TGTTCTCTGATCGCCTCAGTG*	*CCTGGGATCTGTAATCTGACTCT*
*Sp7*	*170574*	*CCCACCCTTCCCTCACTCAT*	*CCTTGTACCACGAGCCATAGG*
*Alpl*	*11647*	*GCTGATCATTCCCACGTTTT*	*CTGGGCCTGGTAGTTGTTGT*
*Bglap*	*12096*	*TCTGACAAAGCCTTCATGTCCA*	*CGGTCTTCAAGCCATACTGGTC*
*Rankl*	*21943*	*TCACCATTCGGATGAGTCTG*	*ACTTGTGGCTCTGATGTTCC*
*Opg*	*18383*	*CCTGAGGCCCAGCCATTT*	*CTTGGCCCAGCCTCGAT*
*Actb*	*11461*	*CCTTGCCCTGACCACTCTTA*	*ACACTGGGCTGCAATACACA*

## Data Availability

Not applicable.

## Supplementary Materials

The following supporting information can be downloaded at: https://www.mdpi.com/article/10.3390/biomedicines10020404/s1, Figure S1: Complete western blot related to Figure 1; Figure S2: Inhibition of Cdk5 with roscovitine alleviates GC-mediated bone loss by reducing osteoclastogenesis; Figure S3: Cdk5 siRNA knockdown modulates the GC-mediated *Rankl*/*Opg* ratio.
