# Supplementary material for: Inhibition of Cdk5 Ameliorates Skeletal Bone Loss in Glucocorticoid-Treated Mice
Source: Biomedicines. 2022 Feb 8;10(2):404. doi: 10.3390/biomedicines10020404 (PMC8962259; doi:10.3390/biomedicines10020404)
Supplement: Supplementary file 1 [file biomedicines-10-00404-s001.zip › Figure-S1.pdf]

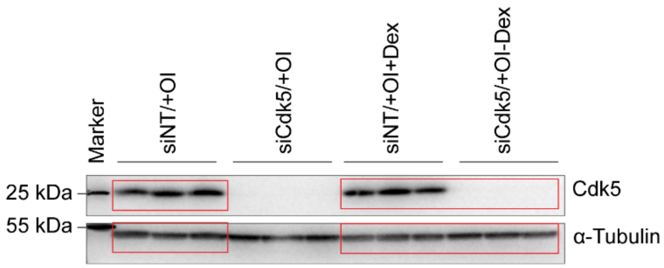

Figure S1. Complete western blot related to Figure 1. Portions that were chosen for presentation are marked in red. The siCdk5/+OI group has been cropped in Figure 1B, as shown with the dotted line.
