# Supplementary material for: Inhibition of Cdk5 Ameliorates Skeletal Bone Loss in Glucocorticoid-Treated Mice
Source: Biomedicines. 2022 Feb 8;10(2):404. doi: 10.3390/biomedicines10020404 (PMC8962259; doi:10.3390/biomedicines10020404)
Supplement: Supplementary file 1 [file biomedicines-10-00404-s001.zip › Figure-S2.pdf]

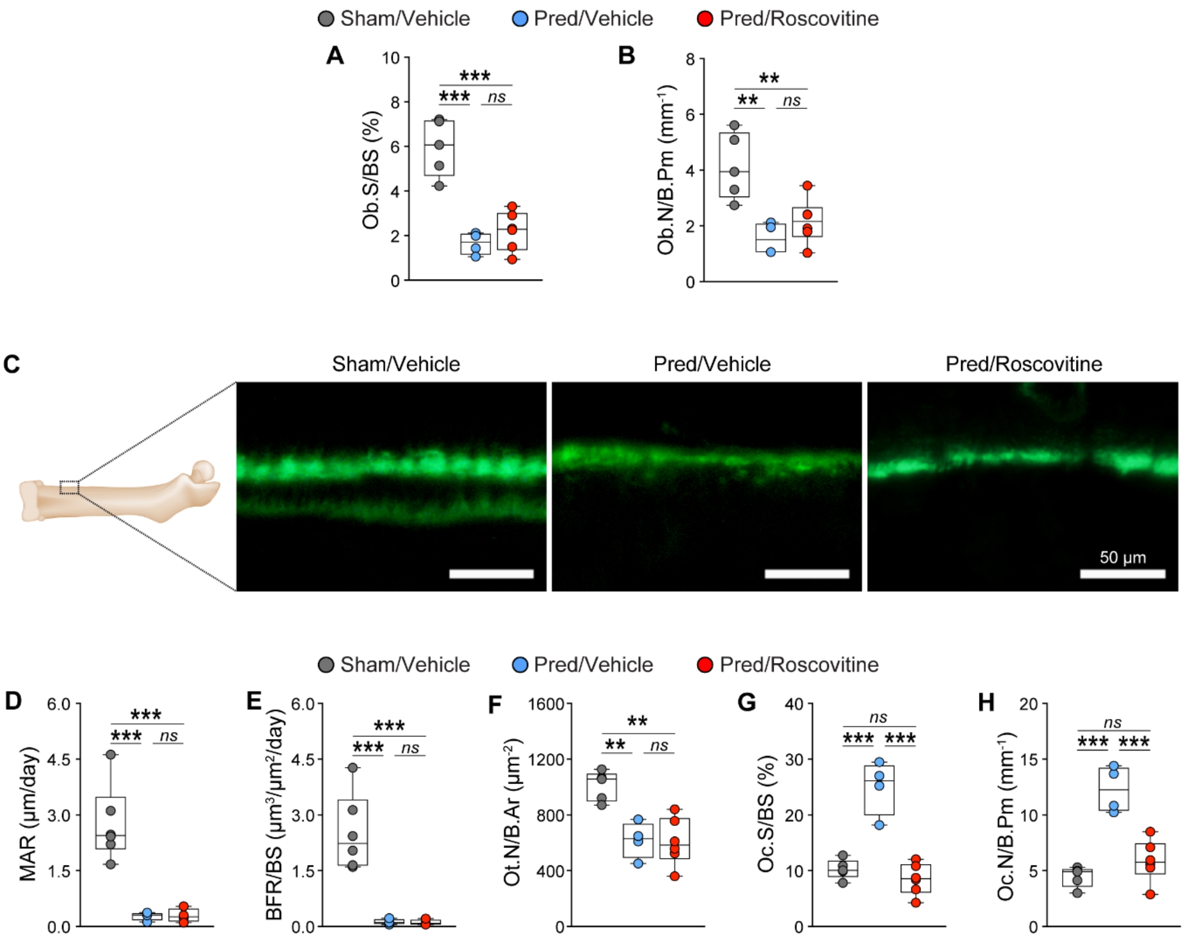

Figure S2. Inhibition of Cdk5 with roscovitine alleviates GC-mediated bone loss by reducing osteoclastogenesis. Static and dynamic bone histomorphometry was performed from different treatments and following parameters were calculated from cortical bone: **(A)**, osteoblast surface per bone surface – Ob.S/BS (%); **(B)**, osteoblast number per bone parameter – Ob.N/B.Pm (mm^{-1}); **(C)**, representative images of dual calcein labeling (green) (scale: 50 µm); **(D)**, mineral apposition rate - MAR (µm/day); **(E)**, bone formation rate - BFR/BS ($\mu m^3/\mu m^2$/day); **(F)**, osteoclast surface per bone surface – Oc.S/BS (%); **(G)**, osteoclast number per bone parameter – Oc.N/B.Pm (mm^{-1}) (n=4-6). Data are represented as box and whisker plots with min. to max. as well as superimposing all of the data points. Statistical differences between two groups were determined by one-way ANOVA and Tukey's test. *$p < 0.05$, **$p < 0.01$, ***$p < 0.001$.
