# Supplementary material for: Inhibition of Cdk5 Ameliorates Skeletal Bone Loss in Glucocorticoid-Treated Mice
Source: Biomedicines. 2022 Feb 8;10(2):404. doi: 10.3390/biomedicines10020404 (PMC8962259; doi:10.3390/biomedicines10020404)
Supplement: Supplementary file 1 [file biomedicines-10-00404-s001.zip › Figure-S3.pdf]

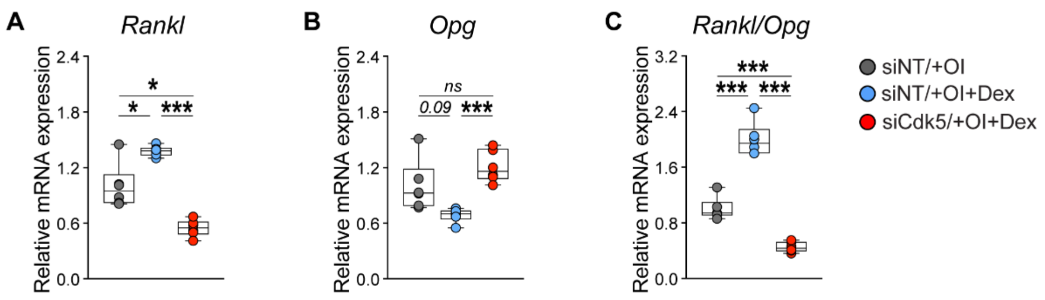

Figure S3. *Cdk5* siRNA knockdown modulates the GC-mediated *Rankl*/*Opg* ratio. Primary murine calvarial osteoblasts were transfected either with non-targeting siRNA (si*NT*) or *Cdk5*-specific siRNA (si*Cdk5*) for eight days in presence or absence of 1 µM dexamethasone (Dex), and mRNA expression of: (**A**), *Rankl*, (**B**), *Opg*, and (**C**), *Rankl*/*Opg* ratio was determined. Data are represented as box and whisker plots with min. to max. as well as superimposing all of the data points. Statistical differences between two groups were determined by one-way ANOVA and Tukey's test. *$p < 0.05$, **$p < 0.01$, ***$p < 0.001$.
